# Supplementary material for: Isolation, NMR Characterization, and Bioactivity of a Flavonoid Triglycoside from Anthyllis henoniana Stems: Antioxidant and Antiproliferative Effects on MDA-MB-231 Breast Cancer Cells
Source: Antioxidants (Basel). 2024 Jun 28;13(7):793. doi: 10.3390/antiox13070793 (PMC11273540; doi:10.3390/antiox13070793)
Supplement: Supplementary file 1 [file antioxidants-13-00793-s001.zip › antioxidants-3070791-supplementary.pdf]

<u>Supplementary file : AA_{770} NMR specta</u>

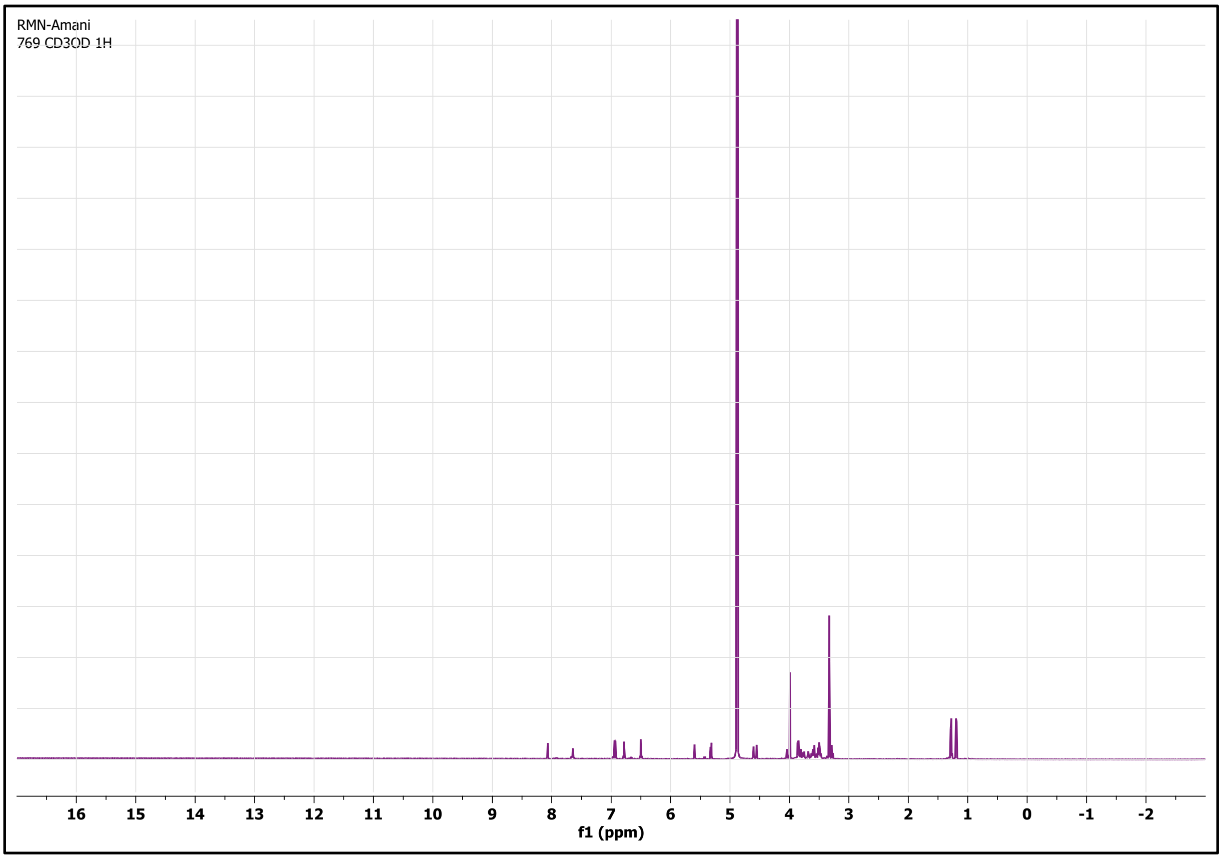

Figure 1: Global NMR 1H Spectrum of compound AA_{770}

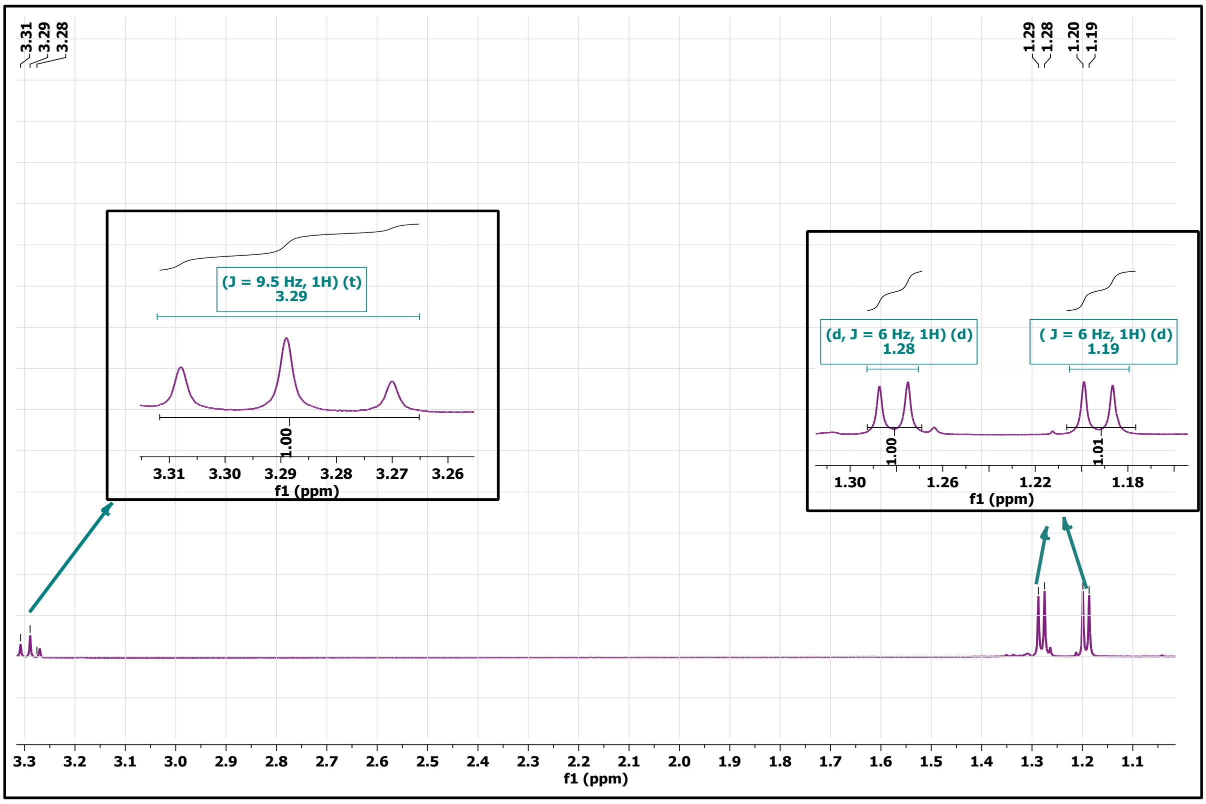

Figure 2: 1H NMR spectrum enlargement from 1.1 to 3.4 ppm of compound AA770 (CD3OD, 500 MHz)

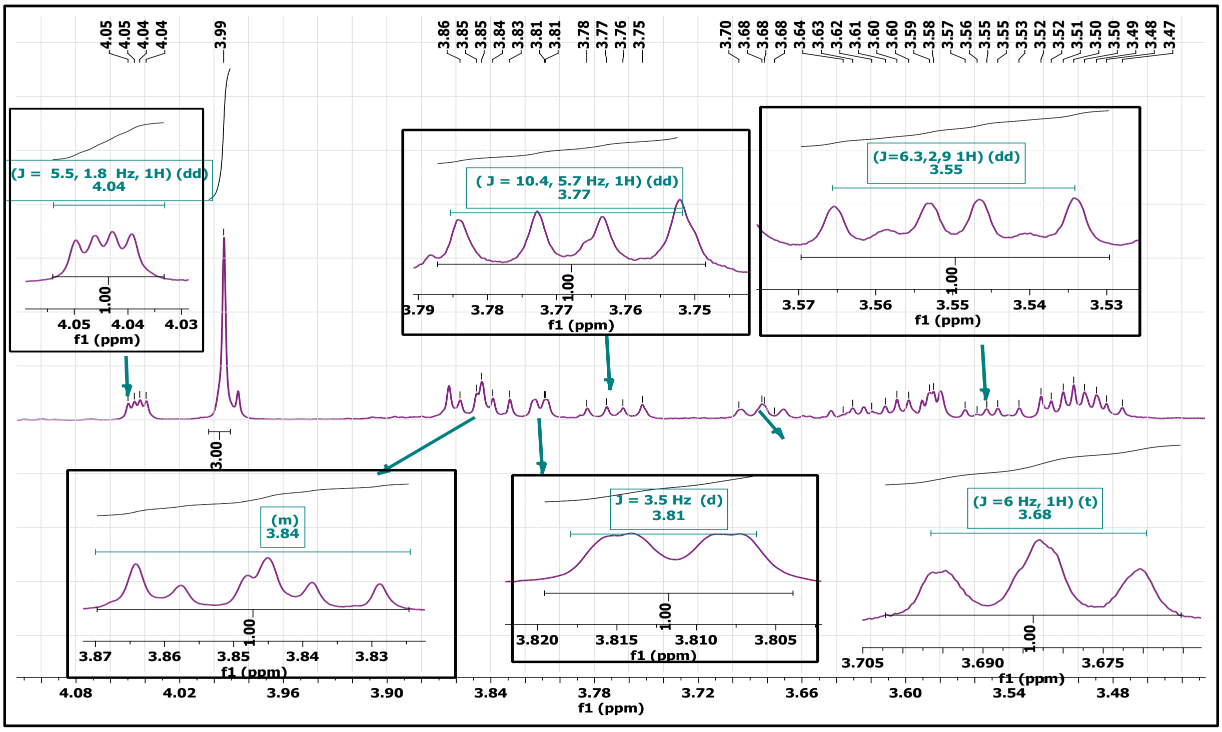

Figure 3: 1H NMR spectrum enlargement from 3.48 to 4.08 ppm of compound AA_{770} (CD_3OD, 500 MHz)

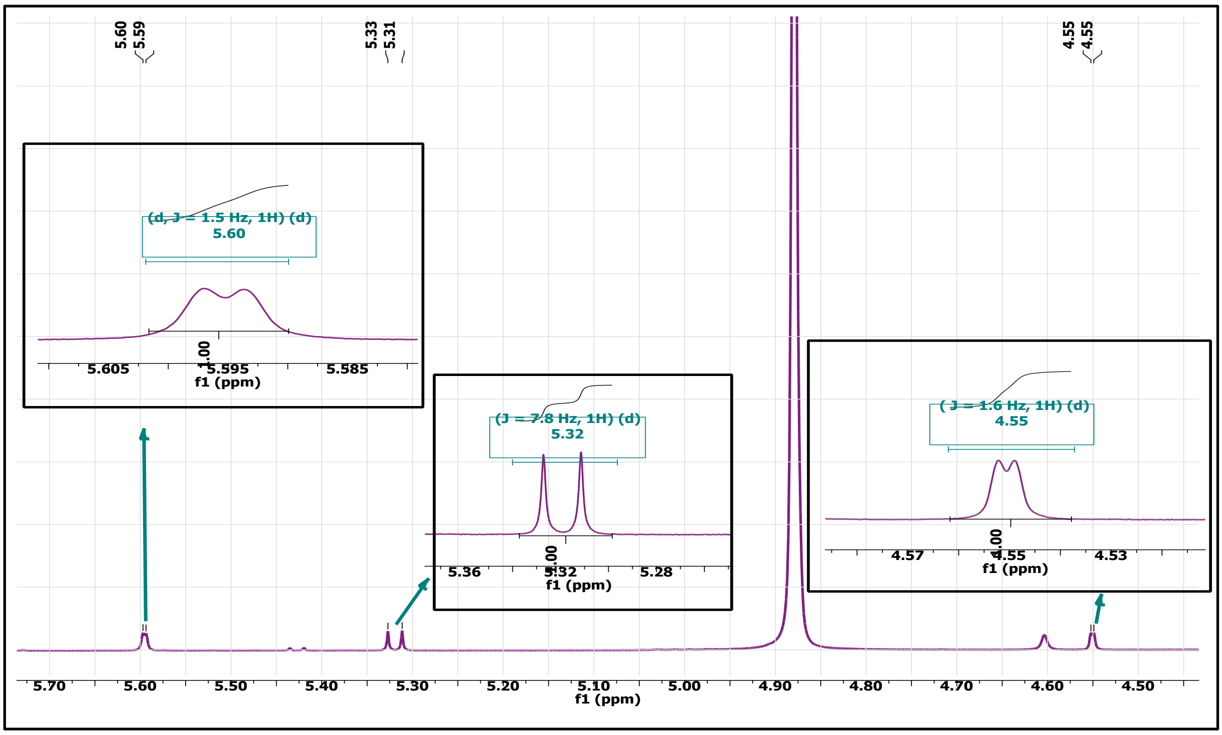

Figure 4: 1H NMR spectrum enlargement from 4.50 to 5.70 ppm of compound AA_{770} (CD_3OD, 500 MHz)

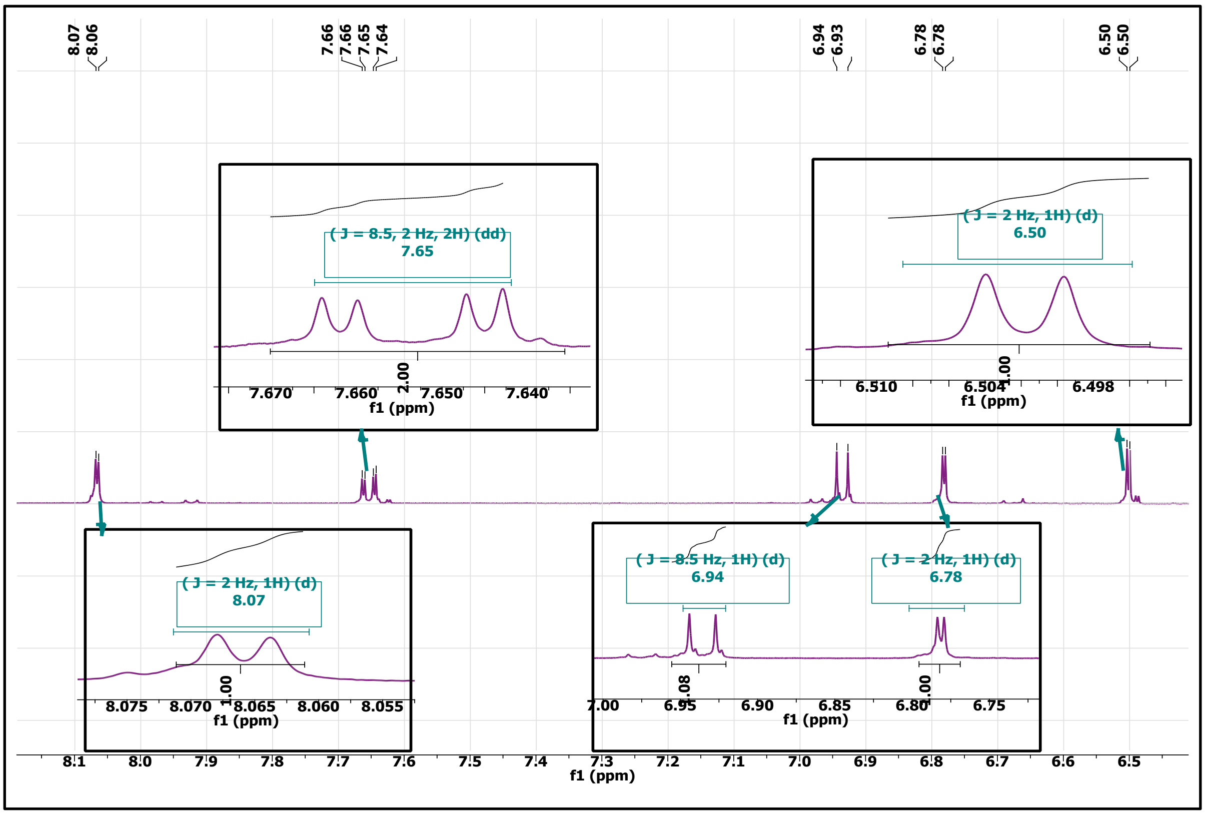

Figure 5: 1H NMR spectrum enlargement from 6.50 to 8.1 ppm of compound AA_{770} (CD_3OD, 500 MHz)

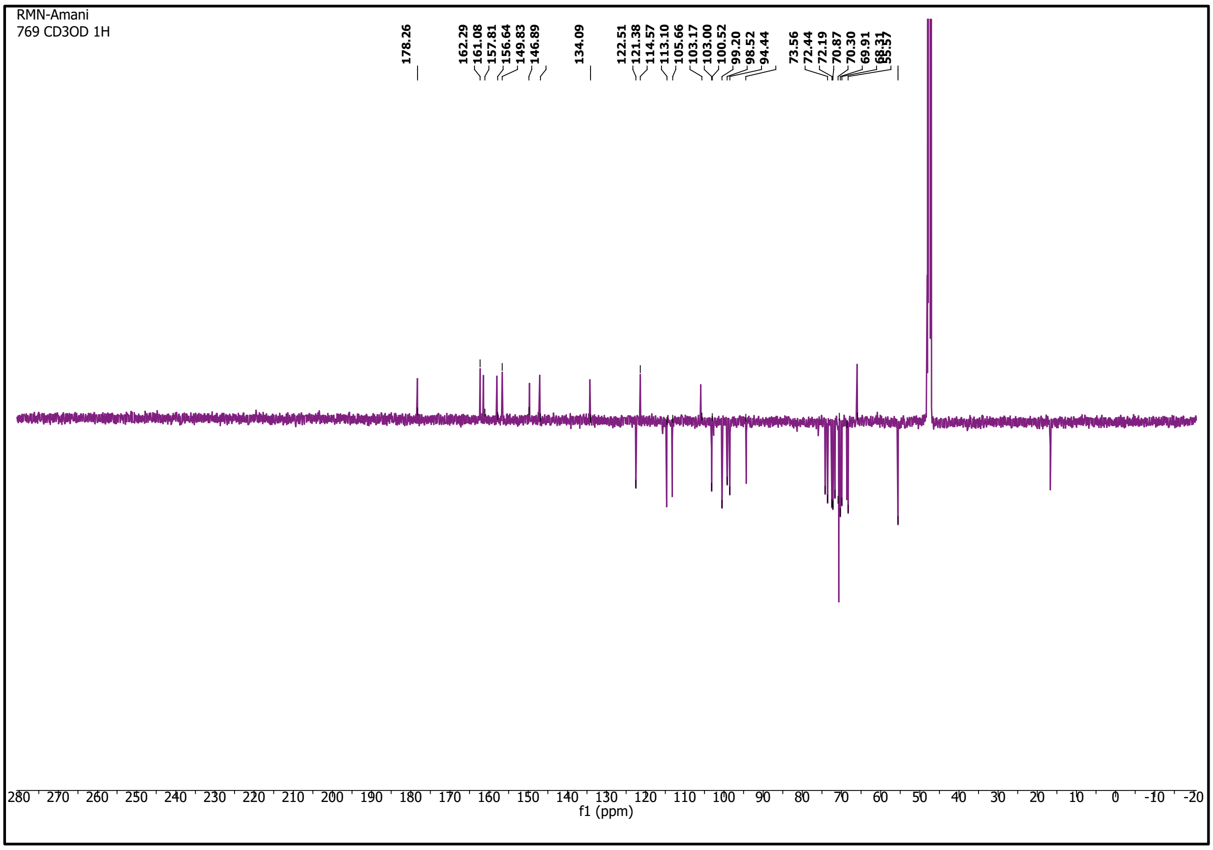

Figure 6: ^{13}C NMR Spectrum of compound AA$_{770}$ (CD_3OD, 125 MHz)

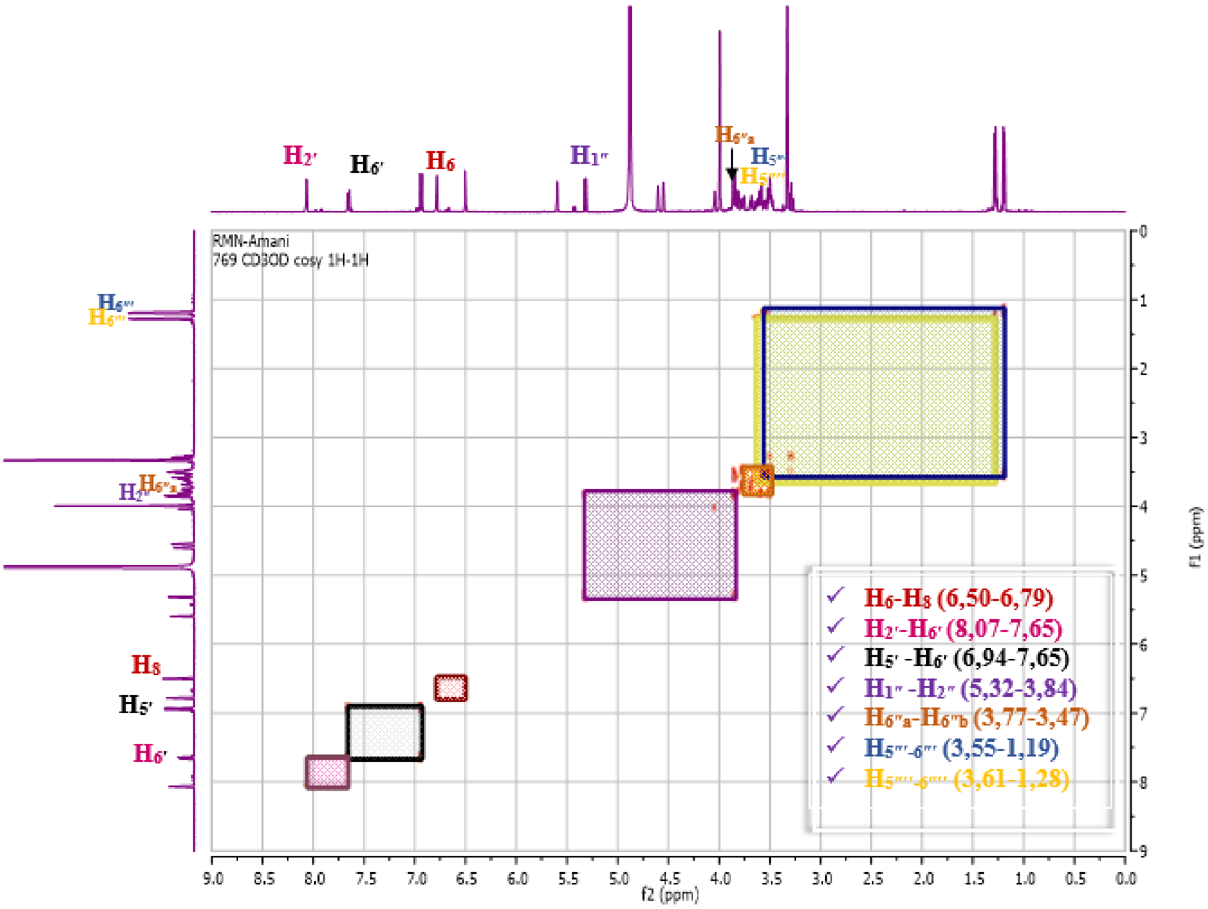

Figure 7: H-H COSY Spectrum of compound AA770

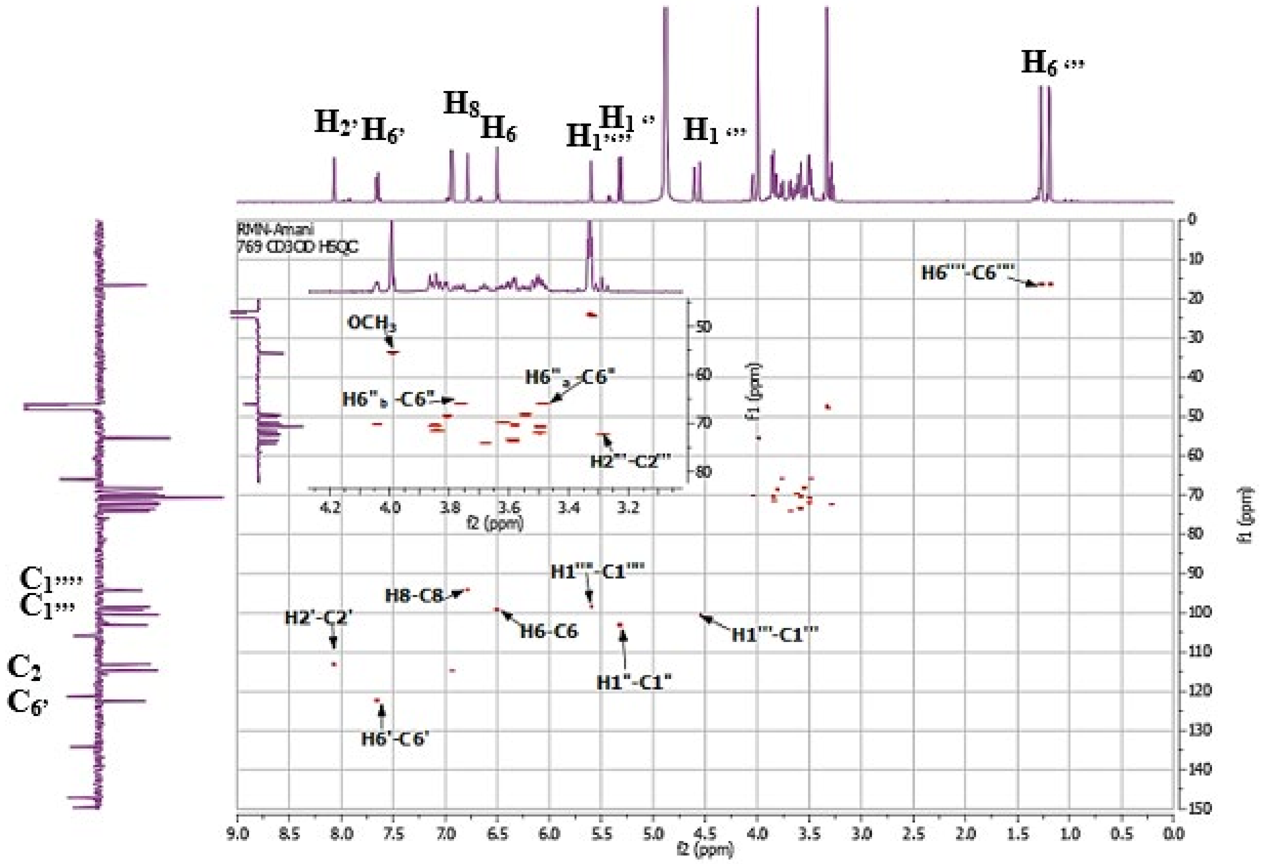

Figure 8: HSQC spectrum of compound AA_{770} and enlargement from 3 to 4.5 ppm

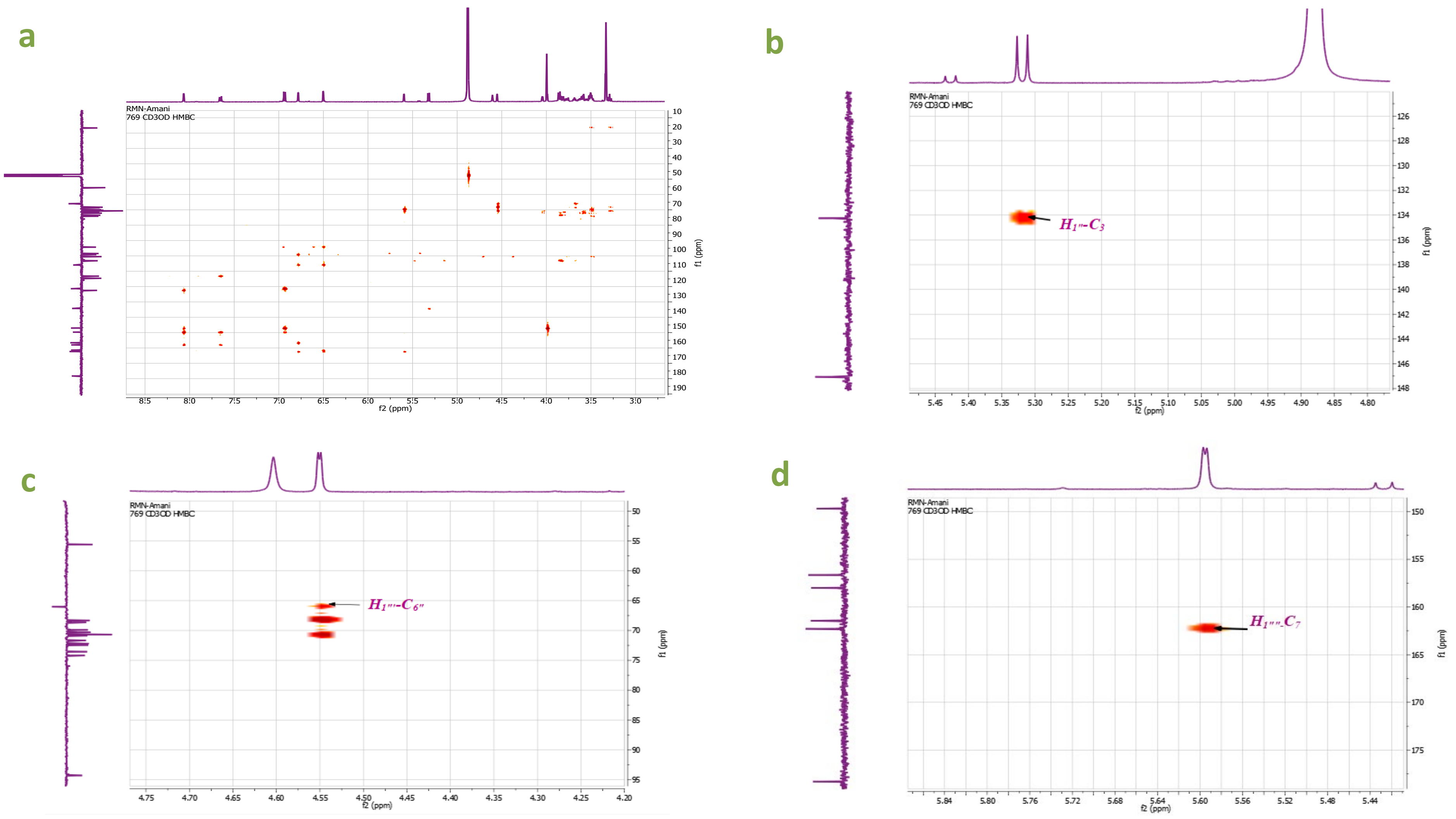

Figure 9: HMBC spectrum of compound AA770 ;a : global spectrum; b :enlargement from 4.80 to 5.45 ppm ; c: enlargement from 4.20 to 4.75 ppm and d : enlargement from 5.45 to 5.84 ppm

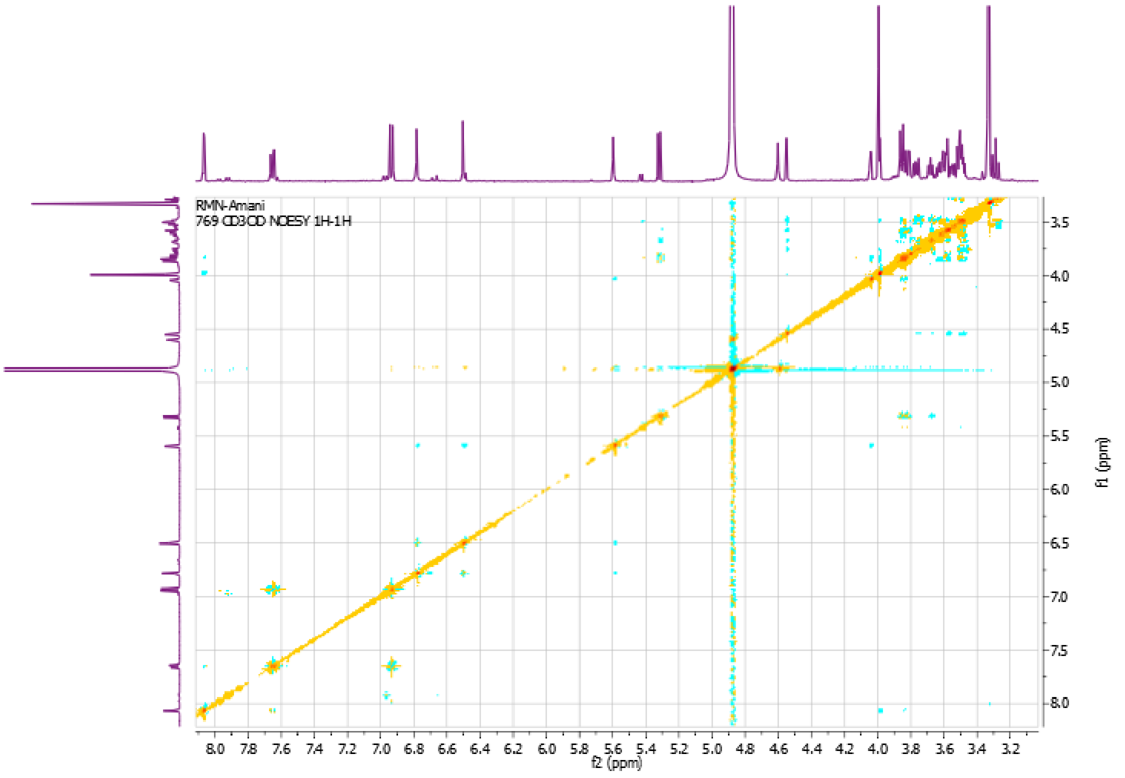

Figure 10: Global NOESY spectrum of compound AA_{770}

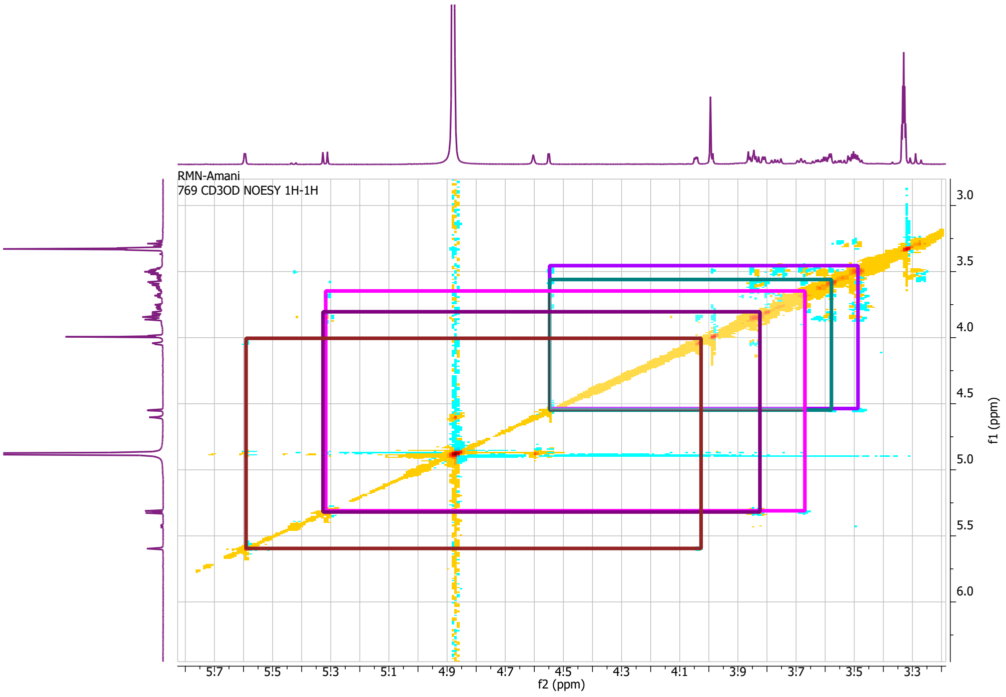

Figure 11: NOESY spectrum of compound AA770: enlargement from 3.3 to 5.7 ppm : we observe correlations between : **$H_{1'''}$ and $H_{5'''}$** (4.55 and 3.55) ; **$H_{1'''}$ and $H_{3'''}$** (4.55 and 3.49) of the second desoxyhexose. **$H_{1''}$ and $H_{5''}$** (5.32 and

3.67) ; **$H_{1''}$ and $H_{3''}$** (5.32 and 3.82) of the hexose. **$H_{1''''}$ and $H_{5''''}$** (5.59 and 4.03) du second désoxyhexose

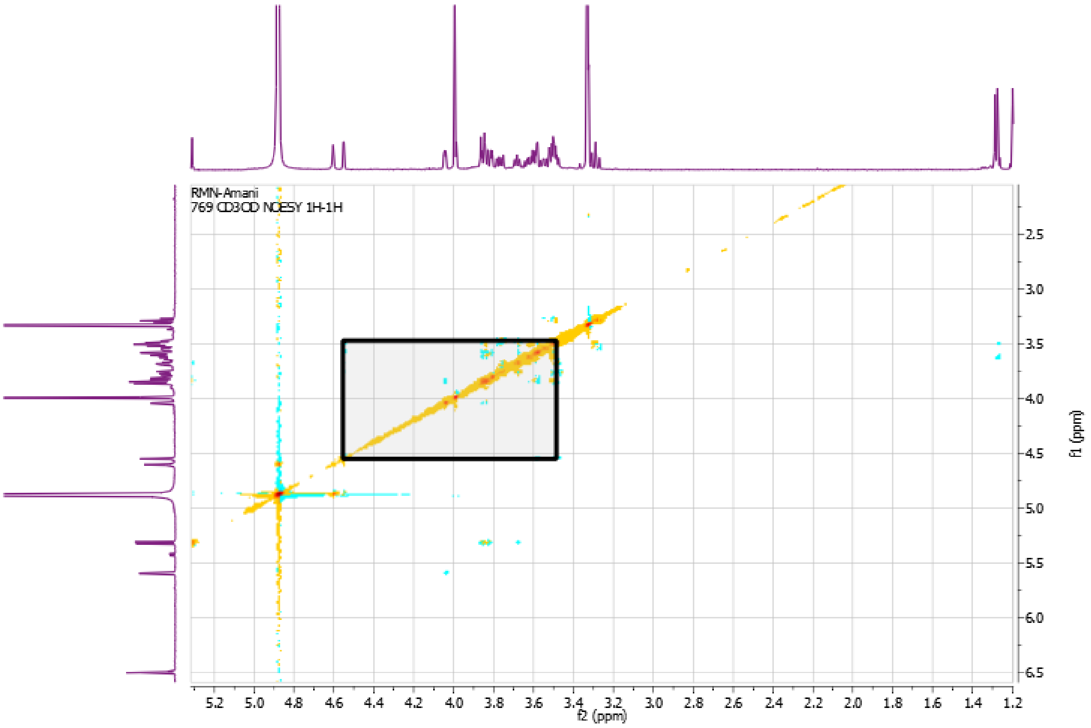

Figure 12: NOESY spectrum of compound AA_{770}: enlargement from 1.2 to 5.2 ppm: we observe correlations between **$H_{6''b}$** and **$H_{1''}$** of the hexose

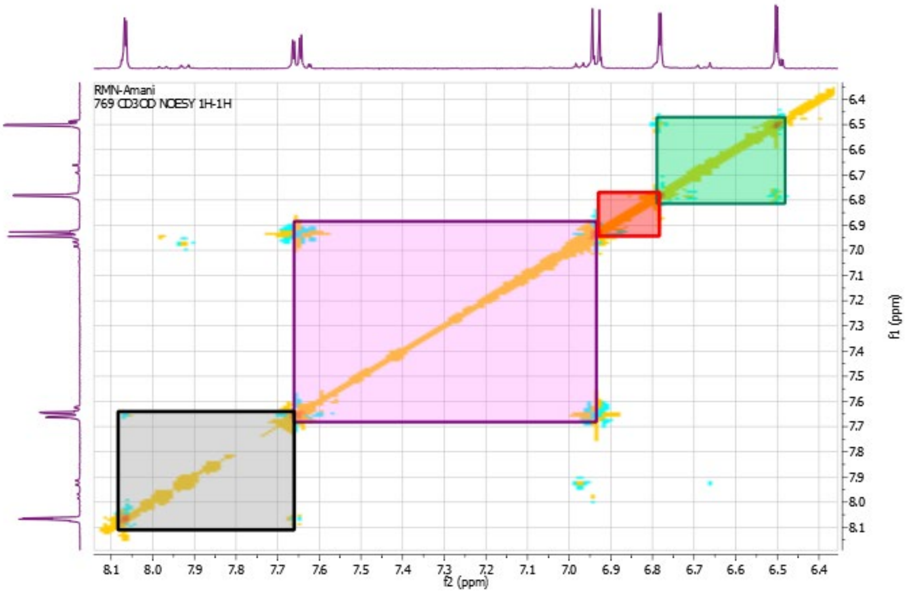

Figure 13: NOESY spectrum of compound AA_{770}: enlargement from 6 to 8.1 ppm: we observe correlations between: **H_8** and **H_6**; **$H_{5'}$** and **$H_{6'}$**; **$H_{2'}$** and **$H_{6'}$**; **H_8** and **$H_{5'}$** (cycle B and cycle A) of the aglycon

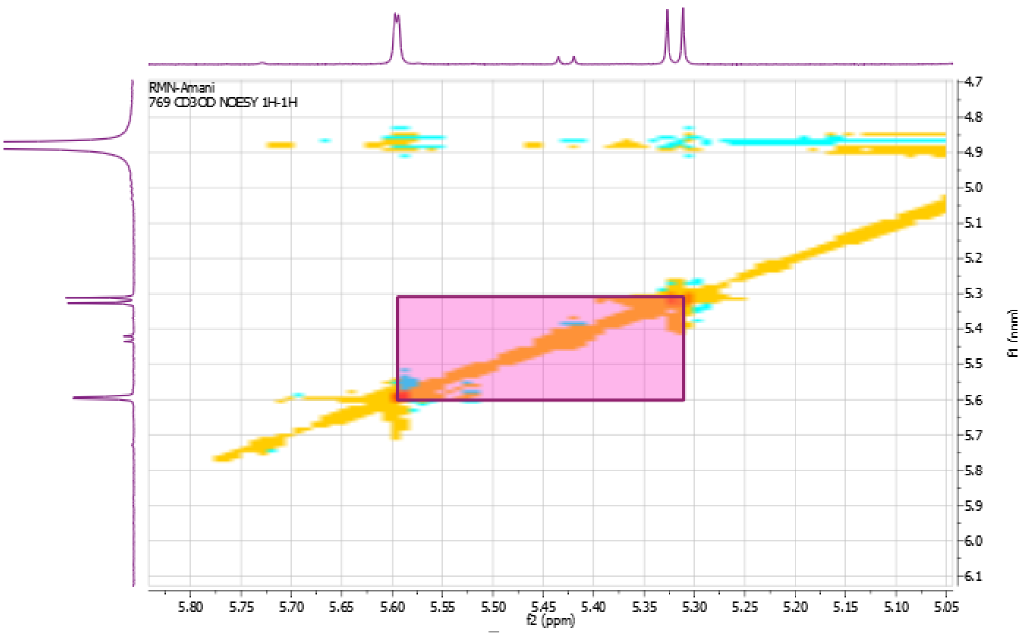

Figure 14: NOESY spectrum of compound AA770: enlargement from 5.05 to 5.80 ppm: we observe correlations between: **$H_{1''}$** and **$H_{1'''}$** of the hexose and its adjacent desoxyhexose

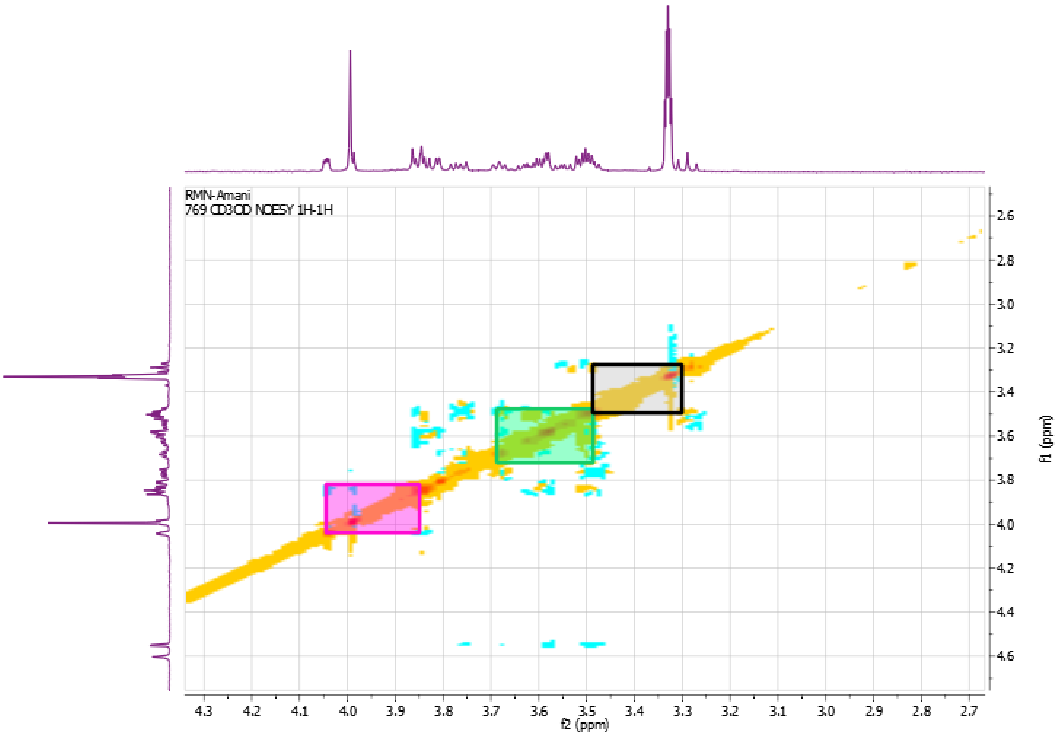

Figure 15: NOESY spectrum of compound AA770: enlargement from 2.7 to 4.3 ppm: we observe correlations between: **$H_{5''}$** and **$H_{3'''}$**, **$H_{4''}$** and **$H_{6''a}$**; **$H_{2'''}$** and **$H_{3'''}$** of the adjacent hexose and the desoxyhexose

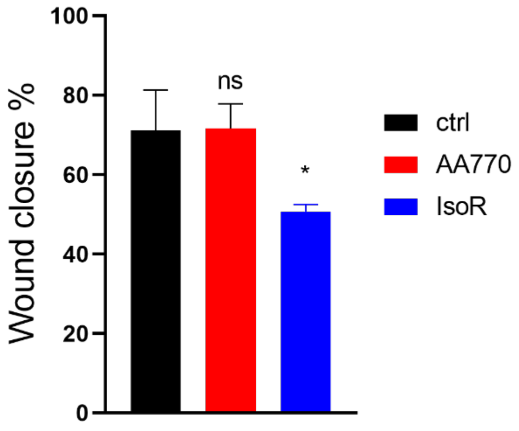

Figure S16: Evaluation of MDA-MB-231 cells migration. Cells were treated with control (DMSO, black bar); 10µmol. L-1 AA770 (red bar) or 10µmol. L-1 isorhamnetin (blue bar). Cell migration was assessed by measuring wound closure after 36 hours with ImageJ software. Results are presented as wound healing percentage (mean ± SE, n = 3, each performed in triplicate. (** $p < 0.01$ Tukey's one-way ANOVA test).
